# Crystallizing the function of the magnetosome membrane mineralization protein Mms6

**DOI:** 10.1042/BST20160057

**Published:** 2016-06-09

**Authors:** Sarah S. Staniland, Andrea E. Rawlings

**Affiliations:** * Department of Chemistry, The University of Sheffield, Brook Hill, Sheffield S3 7HF, U.K.

**Keywords:** iron-binding protein, magnetite nucleation, magnetosome, magnetotactic bacteria, membrane protein, Mms6

## Abstract

The literature on the magnetosome membrane (MM) protein, magnetosome membrane specific6 (Mms6), is reviewed. Mms6 is native to magnetotactic bacteria (MTB). These bacteria take up iron from solution and biomineralize magnetite nanoparticles within organelles called magnetosomes. Mms6 is a small protein embedded on the interior of the MM and was discovered tightly associated with the formed mineral. It has been the subject of intensive research as it is seen to control the formation of particles both *in vivo* and *in vitro*. Here, we compile, review and discuss the research detailing Mms6’s activity within the cell and in a range of chemical *in vitro* methods where Mms6 has a marked effect on the composition, size and distribution of synthetic particles, with approximately 21 nm in size for solution precipitations and approximately 90 nm for those formed on surfaces. Furthermore, we review and discuss recent work detailing the structure and function of Mms6. From the evidence, we propose a mechanism for its function as a specific magnetite nucleation protein and summaries the key features for this action: namely, self-assembly to display a charged surface for specific iron binding, with the curvature of the surfaces determining the particle size. We suggest these may aid design of biomimetic additives for future green nanoparticle production.

## Introduction

Nanoscale inorganic materials are important in an increasingly nanotechnological world. More specifically, magnetic nanoparticles (MNPs) have wide ranging uses from targeted drug delivery [1], to ultrahigh-density data storage [2]. Magnetite MNPs are particularly useful for biomedical applications such as MRI contrast enhancers for diagnostics, magnetically targeted treatments and magnetic hyperthermia therapy [1,3,4]. However, the reliable production of highly specific monodispersed MNP is a considerable challenge making new synthetic routes to precisely tailored MNPs a necessity [3].

Natural organisms carefully control the production of a vast range of inorganic minerals in a process called biomineralization [5,6]. For example organisms use calcium phosphate to form bones and teeth, calcium carbonate to make shells and diatoms produce shells and spines from silica [5,6]. Remarkably, nature offers precise genetic control over mineral formation (down to the nanoscale) using a suite of biomineralization proteins [5,6]. Harnessing these proteins presents a biological (ambient condition) synthetic approach to producing tailored MNPs.

**Figure 1:**
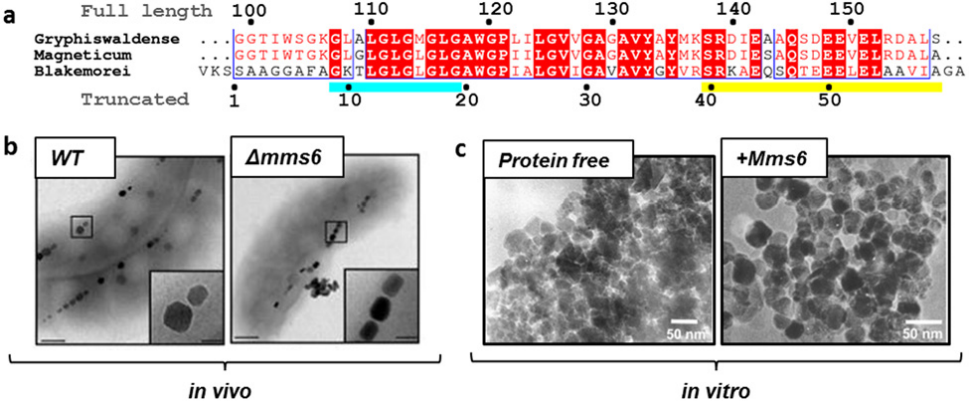
The sequence of Mms6 and its activity demonstrated *in vivo* and *in vitro* (**a**) Sequence alignment of the truncated Mms6 from different MTB species (the full pre-protein amino acid; numbering is shown above and the mature truncated amino acid position for *M. magneticum* AMB-1; Mms6 is shown below the alignment). Conserved residues are highlighted in red boxes and similar residues are in red type, showing a highly conserved truncated protein. The initial approximately 98 residues (assumed absent from the mature protein) are not shown but are less conserved (or missing in the case of *M. blakemorei*). The blue bar highlights the glycine–leucine repeating sequence and the yellow bar highlights the hydrophilic, acid rich, C-terminal amino acid region. (**b**) Demonstrates the activity of Mms6 *in vivo* through an *mms6* knockout mutant in AMB-1 [23]. Note the MNPs formed in the cell with *mms6* absent are smaller and ill formed. (**c**) Demonstrates the activity of Mms6 *in vitro* by comparing magnetite MNPs formed in a simple RTCP (protein-free control) with those formed under the same condition but with the addition of Mms6 [24]. Figures 1(b) and 1(c) reproduced from [24]: Amemiya, Y., Arakaki, A., Staniland, S.S., Tanaka, T. and Matsunaga, T. (2007) Controlled formation of magnetite crystal by partial oxidation of ferrous hydroxide in the presence of recombinant magnetotactic bacterial protein Mms6. Biomaterials **28**, 5381–5389.

The most studied magnetotactic bacteria (MTB) are aquatic, motile, microaerobic microbes that take up soluble iron ions and crystallize magnetite MNPs within intracellular liposomes (magnetosomes) (Figure 1b) [7,8]. However, MTB are found across the phylogenetic tree, leading to bacteria with variable phenotypes. These range from anaerobic to aerobic, micron-sized *Cocci, Vibrio* and *Spirilla* [9]*,* to giant 10 μm *rods* containing 1000’s of magnetosomes [10], living in environments from fresh water to saline [11], whereas some MTB even produce greigite MNP or both magnetite and greigite [12]. Early reviews, such as Bazylinski and Frankel [8] and Frankel et al. [13], offer comprehensive descriptions of MTB and their magnetosomes. Magnetosomes’ size and morphology vary greatly between strains too, but is highly uniform within each strain, demonstrating the control that biomineralization proteins must have over this process. The mechanism of biomineralization in MTB enjoys extensive research and is the subject of several concise overview reviews [7,14,15], and a more specific review of the magnetosomes [16] and of their protein's predicted structure and function [17]. Briefly, the magnetosome membrane (MM) is proposed to form through invagination of the cytoplasmic membrane [18], with recruitment and insertion of unique biomineralization proteins into or on to the membrane [14,18]. These include: iron transporters [19,20], redox proteins [21] that ensure the chemistry of magnetite formation is enabled, and nucleation and shape controlling proteins [22,23] that ensure that magnetite is crystallized and grows in the correct morphology [17]. We have been interested in understanding how these proteins (particularly the latter) control magnetite MNP formation and how we can best utilize them (and their mimics) for bio-mediated MNP formation for applications. There has been considerable analysis of one such protein (magnetosome membrane specific6; Mms6), and this is the subject of this mini-review.

### Mms6 *in vivo*

To understand the molecular elements which control magnetite biomineralization there have been a number of studies of both the genomes and proteomes of different MTB and in particular of those elements closely associated with the formation of the magnetosome itself [18,25–29]. Analysis of these sequences has shown that the majority of genes involved in magnetosome biogenesis can be grouped into four key operons (*mms6, mamGFDC, mamAB* and *mamXY* clusters) in a region of the genome termed the magnetosome island (MAI) [18,28–34]. If the MAI is lost from MTB then the magnetic properties are also lost [31] and vice versa, if these key operons are transferred to non-magnetic bacteria, then they also develop the ability to produce biogenic magnetic nanocrystals [30].

The discovery of Mms6 was reported in 2003 in a pioneering study by Arakaki et al. [35]. The magnetosomes from *Magnetospirillum magneticum* AMB-1 were magnetically extracted from lysed cells and the lipid membrane component of the magnetosome removed [35]. The bare magnetite nanocrystals were subjected to further treatment with detergent and heat to release proteins tightly associated. Four proteins were found: magnetosome membrane specific5 (Mms5), magnetosome membrane specific7 (Mms7), magnetosome membrane specific13 (Mms13) and Mms6, all so-called for their magnetosome membrane specific (Mms) localization and the number denotes their apparent molecular mass [35]. Mms6 has an overall net negative charge at neutral pH, in contrast with the positive charge of the other isolated Mms proteins [35]. Mms6 has been detected in the MM fraction by both 2D SDS/PAGE [25] and shotgun protein identification [18], but not in any of the other membrane fractions of the cell [18], suggesting this protein is specifically targeted to the MM.

The gene sequence of Mms6 is present in several strains of MTB with a high level of consensus, particularly in the C-terminal region (Figure 1a). The sequences code for a protein of approximately 12–15 kDa, much larger than the 6 kDa species isolated directly from magnetosomes [25,35]. The sequence harbours a glycine–leucine repeat motif (cyan in Figure 1a) and an acid rich C-terminal region (yellow in Figure 1a). It is speculated that Mms6 is processed *in vivo* by a specific protease to form the 6 kDa truncated protein (Figure 1a) [25,35]. It is interesting to note that in *Magnetovibrio blakemorei* the *mms6* sequence appears to encode only the truncated form of Mms6 which lacks this N-terminal region [36], which would suggest this part of Mms6 is not required for effective magnetite biomineralization.

A number of gene knockout studies have been performed to assess the specific effect that Mms6 has on magnetite biomineralization [22,23,26]. When the *mms6* gene was knocked out in *M. magneticum* AMB-1, the resulting particles were found to be poorly defined and smaller in size, with an average reduction in diameter of 44% [22] compared with wild-type particles. More recent and previous studies with *Δmms6* strains have shown similar results with approximately 19% reduction in AMB-1 strains [23] (Figure 1b) and a 15% reduction in *Magnetospirillum gryphiswaldense*, indicating Mms6 is required for production of full-sized nanocrystals [26]. Discrepancies in the particle size reduction resulting from *mms6* gene knockouts in different studies may be due to how these knockouts have been performed. The 44% reduction is as a result of an antibiotic resistance cassette insertion in the *mms6* gene, whereas other studies use two-step recombination to delete *mms6*. The cassette insertion could inadvertently result in a more pronounced effect by disrupting the production of downstream gene products which have also been implicated in particle formation [23]. There also appears to be a general loss of shape control when Mms6 is absent [22,23,26]. High resolution transmission electron microscopy (HRTEM) analysis of wild-type nanoparticles shows the presence of cubo-octahedral morphology with the characteristic (100) and (111) crystal faces [22,24,37]. In contrast, a *Δmms6* mutation produces particles bearing the high energy (110) face of magnetite [22], generally considered to be unstable [22]. This may demonstrate the crystallization process is unfinished in this mutated strain. A key phenotype observed in *Δmms6* strains are particles with an elongated morphology (Figure 1b). Wild-type magnetosomes of *M. magneticum* AMB-1 produce particles with a shape factor (ratio of length to width) close to 1, but in *Δmms6* cells this is 0.75 [22,23]. As well as the effects on the nanoparticle itself, the lack of Mms6 in the magnetosome also reduces the level of other magnetosome associated proteins including Mms13, 5 and 7. Mms6 is therefore clearly implicated in protein recruitment to the magnetosome [22], and the N-terminal portion of Mms6 is a likely contender for mediating these contacts.

## Producing MNPs with Mms6 *in vitro*

Additional to its natural activity within the magnetosomes of MTB, purified Mms6 has been investigated in synthetic magnetite formation reactions to look for effects on the MNP products [24,35,38–43]. Magnetite (Fe_3_O_4_) contains both ferric (Fe^3+^) and ferrous (Fe^2+^) iron in a precise stoichiometric ratio of 2:1. There are several methods of producing magnetite synthetically but most rely on providing (or producing during the reaction) a mixture of iron of both valences and bringing about its subsequent precipitation by raising the pH. By including purified Mms6 to these syntheses, the size, shape and material purity of the resulting nanoparticles can be compared with protein-free nanoparticles prepared under identical conditions [24,35,38,40]. The nature of the magnetite precipitation process within the magnetosome has not been completely chemically resolved and remains one of the key barriers to our fuller understanding of magnetite biomineralization. However, a recent well conducted study has gone some way to answering this point [44]. Firlar et al. [44] used single particle analysis of forming magnetosomes to show that a ferric-rich amorphous precursor is formed initially, before conversion to the final magnetite species. It is therefore likely that the current approaches for studying Mms6 activity synthetically share similarities with the processes and conditions under which Mms6 would normally function (Table 1).

**Table 1:** Summary of MNPs produced in Mms6 mediated reactions *Brackets denote the ratio of ferric to ferrous ions used.

	Mms6 construct	MNP synthesis type	MNP size (nm)	Size distribution (σ) (nm)	Control size and distribution (nm)	Reference
Mms6 in solution	Mms6	RTCP (1:1)*	20–30	–	10 with a range 1–100	[35]
			21.2	8.3		[24]
	Mms6	RTCP (1:2)*	22.3	5.2	Asymmetric, peak 8.7 with range 1–90	[41]
	His_6_–Mms6		21.9	6.0		
	Mms6	POFHK	42	22.5 (second peak 136±27.8)	Asymmetric, peak 164 with range 1–600	[41]
	Mms6		20.3	3.2	27.5 with range 10–40	[39]
	Mms6		20.7	3.6	35.5±6.4	[24]
	Mms6	POFHN	145	68.8	Bimodal (peak 59 and 400) range 0–600	[41]
Surface immobilized Mms6			86	21	64 ± 26	[45]
	His_8_–Mms6	POFHK	90	15	69 ± 36	[46]
			87	19	60 ± 21	[40]
	Mms6	POFHN	340	54	230 ± 121	[43]
	Mms6	POFHN (dilute)	231	47	154 ± 63	
Mms6 in pluronic gel	His_6_–Mms6	RTCP (2:1)*	30	–		[38]
Mms6 peptide in solution	M6A	POFHK	22.8	3.3	27.5 with range 10–40	[39]
	GLM6A		20.0	3.1		
Surface immobilized Mms6 peptide	Mms6–pep	POFHK	65	30	60 ± 21	[40]

The majority of studies have explored two methods of magnetite synthesis with Mms6: these are the room temperature co-precipitation (RTCP) and partial oxidation of ferrous hydroxide (POFH). In 2003, the first *in vitro* activity of Mms6 was reported by Arakaki et al. [35]. The authors found that addition of Mms6 to an RTCP reaction (Mms6 at 20 μg/ml) resulted in a product which contained mainly magnetite and little alternative iron oxide compared with the protein-free control sample. The alternative iron oxides formed in the control reaction suggests a relatively uncontrolled precipitation reaction occurred in this experimental system. However, this highlights the ability of Mms6 to shift the balance of products towards magnetite in such a reaction. Additionally, the Mms6 prepared particles displayed a narrower size distribution and a cuboidal appearance very similar to that observed in magnetosomes. Similar results have been replicated with lower concentrations of protein [24] (Figure 1c), different ratios of ferrous to ferric iron in the reaction mixture [41], as well as in POFH reactions [24,41] and with Mms6 immobilized on planar surfaces to mimic the magnetosome interior [40]. The general trends (Table 1) are that the addition of Mms6 in solution produces nanoparticles of approximately 21 nm while also reducing their size distribution, regardless of the protein-free particle size population [with the exception of the partial oxidation of ferrous hydroxide with ammonia and hydrazine (POFHN)]. Interestingly, the only instance when Mms6 mediated particles are of a different size is in a particular partial oxidation of ferrous hydroxide with potassium hydroxide (POFHK) reaction where the control MNPs has a huge size distribution [41]. These reactions are very sensitive to a variety of reaction parameters. In this case, the Mms6 MNPs are an average of 42 nm in size, however if the distribution is examined more closely it can be seen there is a dual population, with one peak the same size as the other Mms6 mediated MNPs (22.5 nm) and the remaining peak similar to the control particles (Table 1) [41], showing Mms6 is able to control a portion of the population, perhaps suggesting the ratio of Mms6 to iron is insufficient. Significantly, on surfaces the particles produced with Mms6 are approximately 90 nm and therefore much larger than the protein-free particles, perhaps due to the planar arrangement of Mms6 compared with the curved micelles in solution. During POFH reactions, the system is heated (to approximately 80°C), at which point most proteins would be denatured and inactive, yet Mms6 has an effect in such reactions. However, the heating is performed once all the reagents are supplied and the particles have begun to precipitate, indicating that Mms6 may either only function at the particle nucleation stage (prior to heating), or be remarkably resilient to such treatment.

## Mms6 *in vitro*: understanding its function

It is clear from the previous section that Mms6 is able to control the formation of magnetite MNPs when added to a chemical precipitation. Taken together we believe we can describe the action of Mms6 *in vitro* and thus propose a mechanism for its function.

### Self-assembly

**Figure 2:**
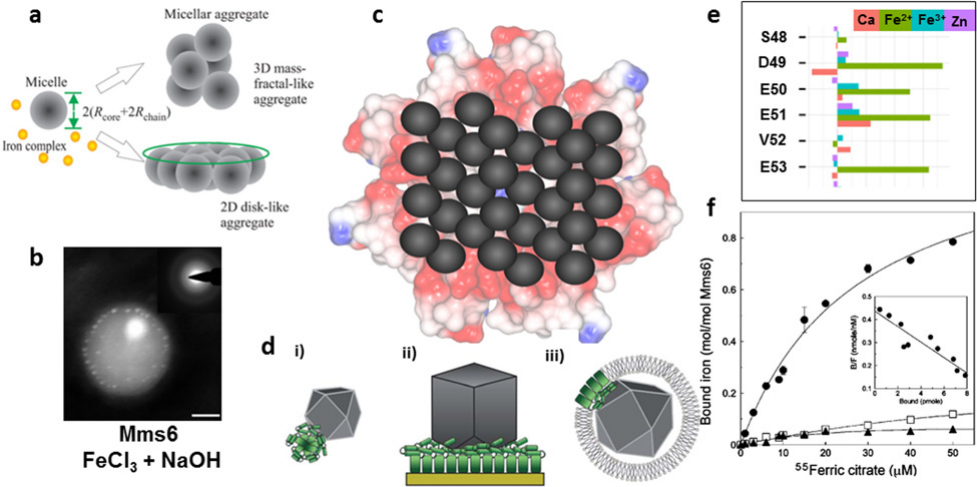
Summary of the self assembly and iron binding properties of Mms6 along with schematic representations of the proposed function Summary of the research on the (1) micellar structure (**a**) and (**b**), (2) schematic representations to describe its function and mechanism for nucleation (**c**) and assembly (**d**) and (3) iron binding (**e**) and (**f**) of Mms6. (**a**) shows a model for the micelle structure obtained by SAXS analysis [48]. (**b**) shows the nucleation and precipitation of iron oxide MNP (bright spots) on the surface of an Mms6 micelle (scale bar 20 nm) [49]. (**c**) An above view schematic of how Mms6 may self-assemble as a protein raft to display regular binding sites for iron ions to nucleate magnetite formation. (**d**) Side-on schematic to demonstrate how the curvature of the protein surfaces differs for the (**i**) in solution micelles, (**ii**) on a surface and (**iii**) on the MM to explain for difference seen in particle size [40]. (**e**) Size of chemical shifts of residues upon metal binding in Mms6 C-terminal peptide from 2D NMR analysis. Green bars represent ferrous ions [51]. (**f**) Ferric iron binding analysis of Mms6 (●), Mms6 with the C-terminus shuffled (▲) and Mms6 with just the acidic residues in the C-terminus shuffled (□) [47]. Figure 2(a) reproduced from [48]: Zhang, H., Liu, X., Feng, S., Wang, W., Schmidt-Rohr, K., Akinc, M., Nilsen-Hamilton, M., Vaknin, D. and Mallapragada, S. (2015) Morphological transformations in the magnetite biomineralizing protein Mms6 in iron solutions: a small-angle X-ray scattering study. Langmuir **31**, 2818–2825. Figure 2(b) reproduced from [49]: Kashyap, S., Woehl, T.J., Liu, X., Mallapragada, S.K. and Prozorov, T. (2014) Nucleation of iron oxide nanoparticles mediated by Mms6 protein *in situ*. ACS Nano **8**, 9097–9106. Figure 2(d) reproduced from [40]: Bird, S.M., Rawlings, A.E., Galloway, J.M. and Staniland, S.S. (2016) Using a biomimetic membrane surface experiment to investigate the activity of the magnetite biomineralisation protein Mms6. RSC Adv. **6**, 7356–7363. Figure 2(e) reproduced from [51]: Rawlings, A.E., Bramble, J.P., Hounslow, A.M., Williamson, M.P., Monnington, A.E., Cooke, D.J. and Staniland, S.S. (2016) Ferrous iron key to Mms6 magnetite biomineralisation: a mechanistic study to understand magnetite formation using pH titration and NMR. Chem. Eur. J. **22**, doi:10.1002/chem.201600322. Figure 2(f) reproduced from [47]: Wang, L., Prozorov, T., Palo, P.E., Liu, X., Vaknin, D., Prozorov, R., Mallapragada, S. and Nilsen-Hamilton, M. (2012) Self-assembly and biphasic iron-binding characteristics of Mms6, a bacterial protein that promotes the formation of superparamagnetic magnetite nanoparticles of uniform size and shape. Biomacromolecules **13**, 98–105.

The amphiphilic nature of Mms6 (Figure 1a) implies it will form micelles in aqueous solution with the C-terminal hydrophilic regions exposed, shielding the hydrophobic N-terminal regions within the core. This structure was first quantitatively investigated by Wang et al. [47] who found through size-exclusion chromatography that the micelle was between 200–400 kDa made up of 20–40 protein subunits. Dynamic light scattering (DLS) measurements found micelles were 10.2 ± 3 nm across, equating to approximately 200 kDa, in agreement with the other analysis [47]. At pH 7.5, they have a slightly narrower elution profile than those at pH 3. The structure and size was further confirmed through SAXS experiments [48]. Using a core-corona model, the data fitted well to a hydrophobic core of radius 3.9 ± 0.4 nm and hydrophilic corona radius of 1.1 ± 0.2 nm parameters at pH 3. Again there is a difference at pH 7.5, but the modelled parameters are not given. Interestingly, they found that addition of iron caused the micelles to form higher order structures such as discs of micelles [48] (Figure 2a) presumably through iron cross-linking. Most recently, Mms6 micelles (or larger proteinaceous assemblies) have been visualized *in situ* in fluid cell TEM [49] where the micelles appear approximately 10-fold larger (Figure 2b). Wang et al. [47] indicated a small population of much larger protein assembly particles in SEC, so it is not unreasonable to assume these will be the easiest to visualize in the fluid cell TEM. Perhaps trace iron is causing a small amount of larger assembly.

It is remarkable that Mms6 is able to convey similar activity/control over magnetite MNP formation *in vitro* as *in vivo,* leading to the conclusion that there must be some degree of self-assembly in the membrane environment, similar to the aggregation seen *in vitro*. We propose Mms6 is not monomeric in the MM, but self-assembles to form protein rafts on the MM interior, displaying a charged C-terminal surface (schematic Figure 2c) akin to the surface of *in vitro* micelles (but of the opposite curvature) (schematic Figure 2d). We tested this hypothesis by enabling Mms6 to self-assemble on a surface, mimicking the membrane environment. Remarkably, the biomimetic Mms6 surface nucleated and controlled magnetite formation, whereas the C-terminal peptide alone (C20Mms6) did not [40]. C20Mms6 is missing the N-terminal region but is still able to assemble on the surface [40]. Thus, we propose that the nature of self-assembly in Mms6 is more specific than generic hydrophobic interactions. A glycine–leucine repeating sequence is present in the conserved 6 kDa protein (but absent from C20Mms6) (Figure 1a). Such motifs are common in self-assembling structural proteins e.g. silk fibroin [40,50]. We propose this knob and hole arrangement of hydrophobic residues could interlock with adjacent Mm6 molecules (and even *in vivo* to other Mms proteins with the same glycine–leucine motif) to form a regularly packed structure and thus regularly space the iron binding C-terminal sites across a raft surface of Mms6 (schematic in Figure 2c). It appears that without this the magnetite nucleation ability is lost, as the C20Mms6 on surfaces demonstrates (compare in Table 1) [40]. In solution, the peptide has shown an effect on particle formation, but this could be driven by some level of aggregation in solution [39]. Interestingly, a peptide constructed of a GL-C6Mms6 repeat fusion (GLM6A) shows better control over particle formation (Table 1), further demonstrating the importance of this region [39].

### Study through the formation process *in situ*

We propose Mms6 self-assembles in a uniform manner to display a regular array of the acidic C-termini, create a negatively-charged surface for iron ion binding to nucleate magnetite formation. However, tracking and analysing this process is not trivial. Recently, Kashyap et al. [49] showed iron oxide nucleation on the surface of the Mms6 micelles *in situ* in a fluid TEM experiment. Remarkably, ferric ion association with Mms6 from low pH can clearly be seen, and as the pH rises small iron oxide particles visibly form across the micelle surface (Figure 2b). They note some ferric ion depletion when the pH initially rises, attributed to a first step in forming a disordered pre-nucleation phase [49]. However, the pH is not quantified for each imaging stage, and only ferric ions are used so the mineral nucleated would not ultimately crystallize to magnetite. Therefore, tracking the chemistry quantitatively throughout the process is essential to understand the effect the protein is having on magnetite formation. We performed a series of pH titrations on the *in vitro* precipitation of magnetite with and without Mms6 and found that Mms6 had no effect on the process below pH 4 for a range of different ferric:ferrous ratios [51]. This is the stage where the more insoluble ferric ions precipitate as a ferric oxide (such as schwertmannite in our case, but could be haematite or ferrihydrite depending on the conditions). Although studies do show that ferric ions can bind to Mms6 at pH 3, it is considerably less than at pH 7 [47]. Thus, we believe binding at low pH is not the main action of Mms6, being negligible when compared with the bulk precipitation. Only after this precipitation stage does the Mms6 pH trace diverge from the protein-free reaction when the mixed valance iron oxides start to precipitate, corroborating the idea that Mms6 is most active at higher pH when the acidic groups are most available for iron binding [51]. Interestingly, we see Mms6 has the most marked effect in ferrous-rich ferric:ferrous ion ratios, indicating increased magnetite production with Mms6 (20%) compared with negligible amounts without protein, suggesting Mms6 is able to direct mineralization towards magnetite synthesis under conditions further from the ideal for magnetite formation, effectively acting as a ‘mineral/ferrous ion buffer’ [51]. Furthermore, it appears that Mms6’s interaction with ferrous ions is potentially crucial to this process.

### Fe binding

The iron binding ability of Mms6 was first reported by Arakaki et al [35]. Using a competitive radioactive ferric ion binding assay where purified recombinant Mms6 was seen to bind Fe^3+^, Ca^2+^ and Mg^2+^, but not Zn^2+^, Ni^2+^ or Cu^2+^, showing some metal selectivity [35]. Most Mms6 iron binding studies have been performed with ferric ions [35,47,49] which have limited solubility at physiological pH. Chelators such as citrate are therefore required to solubilize Fe^3+^ for analysis at neutral pH. High affinity ferric ion binding (*K*_d_=10^−16 ^M) was established at pH 7.5 in this way, whereas mutants (with scrambled C-termini) show no significant ferric binding, demonstrating the importance of the amino acid sequence at the C-termini (Figure 2f) [47]. To facilitate an increase in ferric ion concentration, further assays were performed at pH 3 where ferric ions are soluble. A significantly lower binding affinity of *K*_d_=0.58 ± 0.03 μM was reported [47]. At low pH, the acidic groups of Mms6 are likely to be mostly protonated (p*K*_a_ of free glutamic acid is approximately 4) reducing the capacity to bind ferric ions. As the pH increases to 7 (where mixed valence iron minerals precipitate), deprotonation will produce negatively-charged acidic groups compatible with iron ion binding. Mms6 shows variations in iron ion binding and micelle morphology between low and neutral pH demonstrating the significance that pH has [48]. pH measurements during RTCP with Mms6 further demonstrates this point, showing Mms6 has minimal effect at pH<4 [51]. NMR analysis of the C20Mms6 peptide in the presence and absence of ferric ions (at pH 7) revealed only small chemical shift differences in the peptide side chains [51]. However, in the presence of ferrous ions significant (5-fold) chemical shift differences are seen (Figure 2e) indicating stronger, more specific binding of ferrous than ferric iron [51]. Molecular modelling suggests ferric ions may bind non-specifically (drawn to the areas of greatest charge) so the binding does not significantly change C20Mms6’s conformation, but ferrous ions display specific multisite binding suggesting C20Mms6 is a specific multidentate ferrous ion ligand [51].

## Discussion and conclusion: proposed mechanism of function

*In vitro* Mms6 shows negligible activity in RTCP experiments below pH 5 as determined by pH monitoring *in situ* [51]. When deprotonated the 10–12 nm sized Mms6 micelles are likely to display negatively-charged surfaces for iron binding. We propose that Mms6 binds both ferric and ferrous ions under these conditions; ferrous seemingly specifically [51], whereas the highly charged Fe^3+^ binds more indiscriminately and abundantly [47–49]. The acidic residues of the C-terminal region of Mms6 may concentrate mixed valence iron on the surface in the correct 1:2 ratio to nucleate magnetite. However, the C20Mms6 peptide appears unable to nucleate magnetite as effectively [40], suggesting Mms6 function requires some degree of ordered self-assembly. The structural glycine–leucine repeat sequence may provide this by achieving interlocked packing between Mms6 subunits to bring about a large charged surface for the specific positional binding of Fe^2+^ to 2x Fe^3+^ to encourage the nucleation of magnetite [39,40].

The action of Mms6 *in vivo* may be similar to that observed *in vitro*. Instead of micellar assembly, Mms6 may assemble in the MM in a raft-like form [Figures 2c and 2d (iii)]. Although the pH inside magnetosomes has not been determined, it must be high enough to enable magnetite to precipitate and thus for Mms6 to be active. It is thought that iron is transported into magnetosomes as Fe^2+^ [52–54] with subsequent partial oxidation to Fe^3+^ by oxidase enzymes. In *in vitro* experiments, Mms6 is most influential in ferrous-rich conditions [51], where magnetite is chemically more challenging to produce. This may reflect the conditions within the magnetosome.

Whether or not Mms6 is a nucleating or shape controlling protein is debated [17,22,39,40,49]. Iron ion binding data [47–49] and poor binding activity between magnetite surfaces and Mms6 [40] show it is only tightly bound if involved at the nucleation stages. But *in vivo, mms6* knockout studies show poorly formed, smaller, magnetite crystals, supporting morphology controlling activity [22,23]. However, the more recent studies have found less clear effects on particle morphology when *mms6* is deleted [23]. One possibility to account for these conflicting reports is that neighbouring genes, in particular *mmsF,* may be affected by the gene knockout in the earlier study. MmsF has been described as a master regulator of magnetite biomineralization *in vivo* [23]. However, it is likely that morphology and nucleation activities are coupled; if a particle is not nucleated properly, it may not form to the desired morphology. Equally nucleation from a specific crystal plane will guide the eventual morphology of the final nanoparticle.

It is clear that Mms6 regulates the size of particles *in vitro*; with consistent size across both RTCP and POFHK routes (21 nm) when nucleated on Mms6 micelles in solution [24,35,39,41], and particles approximately 90 nm in size when nucleated by Mms6 assembled on planar surfaces [40,45,46], whereas MNPs within magnetosomes are typically 40–50 nm [55]. The key difference between all these surfaces is curvature, from convex to flat to concave respectively. We suggest this difference in degree and angle of contact between the protein assembly surface and the mineral (along with nucleation physics) is responsible for the difference in particle sizes (Figure 2d and Table 1) [40].

Mms6 activity *in vitro* holds promise for biokleptic synthesis for nanotechnology [56]. However, Mms6 is not trivial to produce, making scale-up for commercial processes unlikely. An understanding of Mms6 informs the design of additives for MNP production to mimic the function of Mms6. We propose that the key elements for design should be: (1) negatively-charged carboxylate-rich surface, (2) a precisely packed assembly of this surface and (3) MNP size may be tuneable by controlling surface curvature.

## Abbreviations

C20Mms6: C-terminus peptide (20 amino acids)
DLS: dynamic light scattering
GLM6A: the most acidic 12 amino acid region of Mms6 C terminus fused to the LG repeat region
HRTEM: high resolution transmission electron microscopy
MAI: magnetosome island
MM: magnetosome membrane
Mms: magnetosome membrane specific
Mms6: magnetosome membrane specific6
MNP: magnetic nanoparticle
MTB: magnetotactic bacteria
POFH: partial oxidation of ferrous hydroxide
POFHK: partial oxidation of ferrous hydroxide with potassium hydroxide
POFHN: partial oxidation of ferrous hydroxide with ammonia and hydrazine
RTCP: room temperature co-precipitation
SEC: size exclusion chromatography
